# Protocol for lentiviral engineering and multi-omic characterization of human kidney tubuloids

**DOI:** 10.1016/j.xpro.2026.104362

**Published:** 2026-02-12

**Authors:** Giulia Perticari, Maroussia M.P. Ganpat, Jarno Drost

**Affiliations:** 1 Princess Máxima Center for Pediatric Oncology, Oncode Institute, Heidelberglaan 25, Utrecht 3584 CS, the Netherlands; 2 Utrecht University, Division Cell Biology, Metabolism & Cancer, Department Biomolecular Health Sciences, Faculty of Veterinary Medicine, Utrecht 3584 CL, the Netherlands

**Keywords:** Cell Biology, Single Cell, Cancer, Genomics, Stem Cells, Organoids

## Abstract

Organoids derived from normal human tissue have proven to be effective preclinical models for studying tumor progression through the introduction of driver mutations. In this protocol, we outline techniques for disease modeling using organoids derived from healthy kidney tissue, also known as tubuloids. We provide step-by-step instructions for the establishment and passaging of tubuloids, lentiviral transduction, processing for histological analysis, and preparation for CUT&RUN and single-cell transcriptomic profiling.

For complete details on the use and execution of this protocol, please refer to Ganpat et al.^1^

## Highlights

• Protocol for establishment, passaging, and lentiviral transduction of tubuloids
• Steps for histological and immunohistochemical staining
• Preparation for CUT&RUN and single-cell transcriptomic profiling
• Applicable to organoids derived from healthy and tumor tissues

## Abstract (editor highlights)

Publisher’s note: Undertaking any experimental protocol requires adherence to local institutional guidelines for laboratory safety and ethics.

## Graphical abstract

**Figure:**
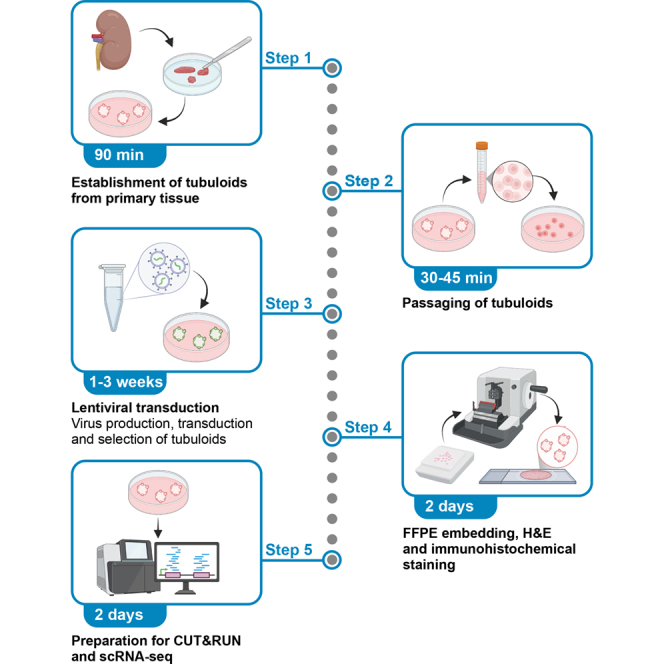

## Teaser

Organoids derived from normal human tissues have proven to be effective preclinical models for studying tumor progression through the introduction of driver mutations. In this protocol, we outline techniques for disease modeling using organoids derived from healthy kidney tissue, also known as tubuloids. We provide step-by-step instructions for the establishment and passaging of tubuloids, lentiviral transduction, processing for histological analysis, and preparation for CUT&RUN and single-cell transcriptomic profiling.

## Before you begin

The following protocol provides a detailed framework for utilizing organoid systems in disease modeling. As a representative example, we employ human kidney-derived organoids, also referred to as tubuloids.^1^^,^^2^^,^^3^ These methodologies are broadly applicable and can be readily adapted to organoid models derived from both normal and tumor tissue samples.

It is preferable to establish tubuloids from freshly isolated primary tissue, processed on the same day as resection. However, in the absence of fresh material, tubuloids can be derived from viably frozen tissue.

### Innovation

This protocol introduces significant improvements over existing approaches.

First, it consolidates into a single workflow all steps required to (1) establish and maintain tubuloids, (2) perform genetic engineering via lentiviral transduction, and (3) carry out comprehensive characterization using histology, CUT&RUN, and single-cell RNA sequencing.

Second, it is specifically optimized for tubuloids, addressing practical challenges such as limited cell numbers and the need to preserve 3D architecture during fixation, while also incorporating standard procedures (e.g., virus production) to ensure completeness.

Finally, the workflow is easily adaptable to other organoid systems, offering a reproducible and comprehensive framework that can serve as a reference for modeling tumorigenesis across diverse disease contexts.

### Institutional permissions

Approval for the use of human material was granted by the medical ethical committee of the Princess Máxima Center for pediatric oncology (Utrecht, the Netherlands). Written informed consent was obtained by all patients and/or their parents/guardians.

Users should obtain all necessary permissions and ethical approvals in accordance with local regulations.

## Key resources table

**Table:** 

REAGENT or RESOURCE	SOURCE	IDENTIFIER
**Biological samples**		

Patient-derived tubuloids	Princess Máxima Center for Pediatric Oncology	N/A

**Chemicals, peptides, and recombinant proteins**		

Advanced DMEM/F12	Thermo Fisher Scientific	Cat# 12634010
Glutamax	Gibco	Cat# 35050061
Hepes	Gibco	Cat# 15630106
Penicillin/Streptomycin	Gibco	Cat# 15140163
B27 supplement	Thermo Fisher Scientific	Cat# 17504044
R-spondin conditioned medium	Produced and tested in-house as described by Pleguezuelos-Manzano et al.^4^	N/A
N-acetylcysteine	Sigma-Aldrich	Cat# A9165
Primocin	InvivoGen	Cat# ant-pm-1
RhoKinase inhibitor Y-27632	Abmole Bioscience	Cat# M1817
EGF	PeproTech	Cat# AF-100-15
FGF10	PeproTech	Cat# 100-26
A83-01	Tocris	Cat# 2939/10
Next Generation Surrogate (NGS) Wnt	ImmunoPrecise Antibodies	Cat# N001 - 100 mg
BME	Trevigen	Cat# 3533-010-02
TrypLe Express	Thermo Fisher Scientific	Cat# 12605010
Collagenase	Sigma-Aldrich	Cat# C9407
Trypan blue Solution	Thermo Fisher Scientific	Cat# 15250061
Red Blood Cell Lysis Buffer	Roche	Cat# 11814389001
DMEM (1X) + Glutamax	Gibco	Cat# 31966-021
Dulbecco’s phosphate-buffered saline (DPBS) 1X	Gibco	Cat# 14190144
Fetal Bovine Serum (FBS)	Sigma-Aldrich	Cat# F0804
OptiMEM	Gibco	Cat# 31985070
Polyethylenimine, Linear, MW 25000, Transfection Grade (PEI 25K)	Tebu-Bio	Cat# 23966-100
Polybrene	Santa Cruz Biotechnology	Cat# SC-134220
Paraformaldehyde 16% (w/v) in aqueous solution methanol-free	VWR	Cat# 43368.9M
Butanol	Avantor	Cat# 20808.325
Ethanol (absolute)	Boom	Cat# 84028185.2500
Xylene	Boom	Cat# 760518181000
Hematoxylin	Epredia	Cat# 7211
Eosin	Epredia	Cat# 6766007
Bovine Serum Albumin solution (BSA)	Sigma-Aldrich	Cat# A8412
Phosphate-buffered saline (PBS) tablets	Gibco	Cat# 11510546
Citric acid monohydrate	Thermo Fisher Scientific	Cat# 022869.A1
Disodium hydrogen phosphate-2 hydrate	Sigma-Aldrich	Cat# 71643
Sodium azide	Sigma-Aldrich	Cat# 1.06688
Hydrogen peroxide 30%	VWR	Cat# 23622.298
Trisodium citrate-2-hydrate	Sigma-Aldrich	Cat# S4641-1KG
Tris Base	Sigma-Aldrich	Cat# T1699-100ML
Hydrochloric acid	Acros Organics	Cat# 124620010
Citric acid monohydrate	Thermo Fisher Scientific	Cat# 022869.A1
3,3′-Diaminobenzidine (DAB)	Thermo Fisher Scientific	Cat# H54000.06
Pure spermidine	Sigma-Aldrich	Cat# S2626-1G
Saponin	Sigma-Aldrich	Cat# 47036-50G-F
EDTA	MPBio	Cat# 194822
EDTA-free Protease Inhibitor	Roche	Cat# 11873580001
EGTA	Sigma-Aldrich	Cat# 324626
NP-40	Sigma-Aldrich	Cat# 9016-45-9
Proteinase K	Invitrogen	Cat# AM2548
Sodium chloride	VWR	Cat# 27810.295
ProteinA-MN	Zeller et al.^5^	N/A
Calcium chloride	Sigma Aldrich	Cat# C1016-100G
4′,6-diamidino-2-phenylindole (DAPI)	Thermo Fisher Scientific	Cat# D1306
DRAQ5	BioLegend	Cat# 424101
Hoechst 34580	Thermo Fisher Scientific	Cat# H21486
Pertex	Histolab	Cat# 00801

**Experimental models: Cell lines**		

HEK293T	ATCC	CRL-3216

**Recombinant DNA**		

pHDM-VSV-G (envelop plasmid)	Garcia-Beltran et al.^6^	Addgene Cat# 164440
pRC/CMV-Rev1b (packaging plasmid)	Garcia-Beltran et al.^6^	Addgene Cat# 164443
pHDM-Tat1b (packaging plasmid)	Garcia-Beltran et al.^6^	Addgene Cat# 164442
pHDM-Hgpm2 (packaging plasmid)	Garcia-Beltran et al.^6^	Addgene Cat# 164441

**Other**		

12 well tissue culture plates	Greiner Bio-One	Cat# 665180
6 well tissue culture plates	Greiner Bio-One	Cat# 657160
15 mL tubes	Falcon	Cat# 10773501
50 mL tubes	Falcon	Cat# 10788561
Ultracentrifuge tubes	Beckman Coulter	Cat# 355618
Safe-Lock Tube 1.5 mL	Eppendorf	Cat# EP0030123611
DNA LoBind Tube 1.5 mL	Eppendorf	Cat# 022431021
Protein LoBind Tube 0.5 mL	Eppendorf	Cat# 022431064
100 μm cell strainer	VWR	Cat# 76327-102
70 μm cell strainer	Greiner Bio-One	Cat# 542070
FACS tube (Round bottom tube with cell strainer cap)	Falcon	Cat# 352235
Sterile syringe filter, 0.45 μm	Corning	Cat# 431220
20 mL syringe	BD	Cat# 300629
Disposable scalpels	Swann-Morton	Cat# 0501
Glass Petri dishes	Sigma-Aldrich	Cat# Z740620-24EA
Glass Pasteur pipettes	Duran Wheaton Kimble	Cat# 91704024
Superfrost microscope slides	Epredia	Cat# J1800AMNZ
Microscope Coverslips	Menzel-Gläser	Cat# BB024060A1
Fisherbrand glass vial	Thermo Fisher Scientific	Cat# 11516074
Embedding metal mold	Epredia	Cat# 6401018
Embedding cassette	Epredia	Cat# B851110WH

**Reagent or Resource**		

Shaking incubator	Benchmark Scientific	Cat# H1001-M-E
Rotary microtome	Thermo Fisher Scientific	Cat# HM355S
Vortex-Genie 2 mixer	Scientific Industries	Cat# SI-0236
HistoCore Arcadia H - Heated Paraffin Embedding Station	Leica	Cat# 14039357258

## Materials and equipment

**Table:** HEK293T cells medium

Reagent	Final concentration	Amount
DMEM (1X) + Glutamax	N/A	500 mL
Fetal Bovine Serum (FBS)	10%	50 mL
Penicillin/Streptomycin (10000 U/mL)	100 U/mL	5 mL
**Total**		**555 mL**

Store at 4°C.

**Table:** AdvDF+++ base medium

Reagent	Final concentration	Amount
Advanced DMEM/F12	N/A	500 mL
Hepes (1M)	10 mM	5 mL
Glutamax (100X)	1X	5 mL
Penicillin/Streptomycin (10000 U/mL)	100 U/mL	5 mL
**Total**		**515 mL**

Store at 4°C.

**Table:** Organoid culturing medium

Reagent	Final concentration	Amount
AdvDF+++	N/A	43.87 mL
A83-01 (5 mM)	5 μM	50 μL
B27 supplement (50X)	1.5%	750 μL
EGF (0.5 mg/mL)	50 ng/mL	5 μL
FGF10 (0.1 mg/mL)	100 ng/mL	50 μL
N-acetylcysteine (500 mM)	1.25 mM	125 μL
Primocin (50 mg/mL)	0.1 mg/mL	100 μL
Rho-kinase inhibitor Y-27632 (10 mM)	10 μM	50 μL
R-spondin conditioned medium (100%)	10%	5 mL
Next Generation Surrogate WNT (0.5 μM)	0.5 nM	50 μL
**Total**		**50 mL**

Once prepared, store at 4°C and use within 3 weeks. Prewarm at 37°C for 15 min before use.

***Note:*** Alternatively to in-house produced R-spondin conditioned medium, use 1% of commercially available Rspo3-Fc Fusion Protein Conditioned Medium (ImmunoPrecise Antibodies, IpA#R001, 100% stock concentration).

**Table:** Collagenase solution

Reagent	Final concentration	Amount
AdvDF+++	N/A	9 mL
Collagenase (10 mg/mL)	1 mg/mL	1 mL
Rho-kinase inhibitor Y-27632 (10 mM)	10 μM	10 μL
**Total**		**10 mL**

Prepare fresh and prewarm at 37°C before use.

**Table:** PO-blocking buffer

Reagent	Final concentration	Amount
Citric acid monohydrate	39.1 mM	8.22 g
Disodium hydrogen phosphate-2 hydrate	120.9 mM	21.52 g
Sodium azide	30.8 mM	2.0 g
Demi water	N/A	1 L
Hydrogen peroxide	1.5% (v/v)	add 10 mL to 190 mL of PO-blocking buffer
**Total**		**1 L**

Store at 18°C–25°C for up to 3 months. Add hydrogen peroxide freshly before use.

**Table:** Tris/EDTA (antigen retrieval buffer) pH 9.0

Reagent	Final concentration	Amount
Tris base	10 mM	1.21 g
EDTA	1 mM	0.37 g
Demi water	N/A	1 L
Hydrochloric acid (1M)	N/A	Adjust buffer pH to 9.0
**Total**		**1 L**

Store at 18°C–25°C for up to 3 months.

**Table:** Citrate buffer (antigen retrieval buffer) pH 6.0

Reagent	Final concentration	Amount
Trisodium citrate-2-hydrate	10 mM	2.94 g
Demi water	N/A	1 L
Citric Acid (1M)	N/A	Adjust buffer pH to 6.0
**Total**		**1 L**

Store at 18°C–25°C for up to 3 months.

**Table:** DAB buffer pH 5.8

Reagent	Final concentration	Amount
Citric acid monohydrate	39.6 mM	8.32 g
Disodium hydrogen phosphate-2 hydrate	120.9 mM	21.52 g
Demi water	N/A	1 L
Citric acid (1M)	N/A	Adjust buffer pH to 5.8
**Total**		**1 L**

Store at 4°C for up to 6 months.

**Table:** DAB solution 10X

Reagent	Final concentration	Amount
3,3′-Diaminobenzidine (DAB)	28 mM	60 mg
Demi water	N/A	10 mL
**Total**		**10 mL**

Prepare in a fume hood due to the toxicity of DAB. Make 100 μL aliquots and store at −20°C.

Before use, thaw one aliquot and dilute 100 μL of DAB solution 10X with 900 μL of DAB buffer to prepare a 1X working solution.

**Table:** Acidic ethanol

Reagent	Final concentration	Amount
Ethanol (95%)	47.5%	250 mL
Demi water	N/A	250 mL
Hydrochloric acid	0.12% (v/v)	612 μL
**Total**		**500 mL**

Store at 18°C–25°C.

**Table:** Wash buffer stock for CUT&RUN

Reagent	Final concentration	Amount
Hepes (1M)	20 mM	1 mL
Sodium chloride (5M)	150 mM	1.5 mL
Pure spermidine (925 mg/mL)	66.6 μg/mL	3.6 μL
Saponin (10%)	0.05%	250 μL
RNase-free water	N/A	47.25 mL
**Total**		**50 mL**

Prepare fresh before use and keep on ice/at 4°C.

**Table:** Wash buffer 1 for CUT&RUN

Reagent	Final concentration	Amount
Wash buffer stock	N/A	50 mL
EDTA-free protease inhibitor	N/A	1 tablet
EDTA (0.5 M)	2 mM	200 μL
**Total**		**50 mL**

Prepare fresh before use and keep on ice/at 4°C.

**Table:** Wash buffer 2 for CUT&RUN

Reagent	Final concentration	Amount
Wash buffer stock	N/A	50 mL
EDTA-free protease inhibitor	N/A	1 tablet
**Total**		**50 mL**

Prepare fresh before use and keep on ice/at 4°C.

**Table:** Wash buffer 3 for CUT&RUN

Reagent	Final concentration	Amount
Wash buffer stock	N/A	50 mL
**Total**		**50 mL**

Prepare fresh before use and keep on ice/at 4°C.

**Table:** Stop solution

Reagent	Final concentration	Amount
EGTA (0.5 M)	40 mM	8 μL
NP-40 (10%)	1.5%	15 μL
Proteinase K (20 mg/mL)	2 mg/mL	10 μL
RNase-free water	N/A	67 μL
**Total**		**100 μL**

Prepare fresh before use and keep on ice/at 4°C.

**Table:** FACS buffer

Reagent	Final concentration	Amount
Dulbecco’s phosphate-buffered saline (DPBS) 1X	1X	1 mL
Fetal bovine serum (FBS)	0,1%	1 μL
Rho-kinase inhibitor Y-27632 (10 mM)	10 μM	1 μL
**Total**		**1 mL**

Prepare fresh before use and keep on ice/at 4°C.

## Step-by-step method details

### Establishment of human kidney tubuloids from primary tissue

**Timing: 90 min**

This section describes the steps to generate human kidney tubuloids from primary tissue (tumor and/or normal).

1. Collect the tissue in a 50 mL tube filled with 40 mL of AdvDF+++ medium.
2. Place the tissue in a 10 cm glass petri dish and mince it into pieces of ∼1 mm^3^ using two scalpels (Figure 1A).

**Figure 1:**
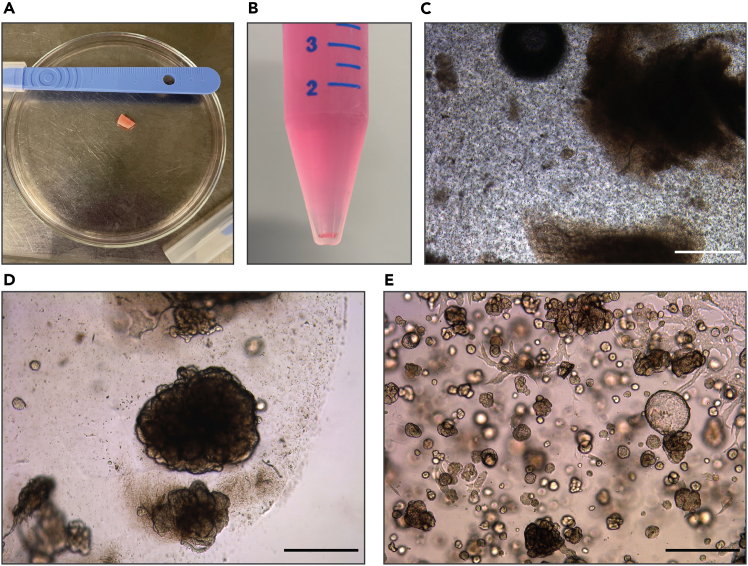
Establishment of human kidney tubuloids from primary tissue (A) Resected primary tissue before mincing. (B) Example of a cell pellet that is obtained after digestion of the tissue with collagenase solution. (C) The digested tissue plated in BME. (D and E) Representative brightfield image of a typical tubuloid culture one week after plating (D) and after the first passage (E). Scale bars = 500 μm.
3. Transfer the minced tissue to a 15 mL tube. Wash the petri dish with 5 mL of AdvDF+++ to collect all the tissue and transfer the suspension to the same tube.
4. Centrifuge the tube at 250 x *g* for 5 min at 4°C and remove the supernatant.
5. Perform tissue digestion with collagenase.
  a. Add 5 mL of pre-warmed collagenase solution (37°C) to the pellet.
  b. Place the tube on a pre-warmed shaking incubator at 250 rpm for 30–45 min at 37°C.
  c. After the initial 15 min of incubation, check if the suspension is homogeneous and if most pieces of tissue have disappeared.

**CRITICAL:** Do not exceed the maximum incubation time of 60 min.

6. After visible tissue dissociation, fill the tube with 10 mL of AdvDF+++ and mix by inverting the tube 10 times.
7. Centrifuge at 250 x *g* for 5 min at 4°C and remove the supernatant (Figure 1B).

***Optional:*** If chunks of tissue are still visible, add 5 mL of AdvDF+++ to the 15 mL tube and filter the suspension through a 100 μm nylon cell strainer. Centrifuge at 250 x *g* for 5 min at 4°C and remove the supernatant.

8. In case of visible red pellet, add 1 mL of red blood cell lysis buffer to the pellet and shake (do not pipet). Incubate for 5 min at 18°C–25°C, then add 10 mL of AdvDF+++. Centrifuge at 250 x *g* for 5 min at 4°C and remove the supernatant.
9. Plate the cell pellet in BME.
  a. Carefully resuspend the pellet by pipetting up and down without creating air bubbles.
  b. Add 70%–75% volume of BME to the pellet.
  c. Resuspend carefully with a p200 pipette and plate 20 μL droplets in a pre-warmed multiwell cell culture plate, based on total plating volume. In 24-well, 3 droplets of 20 μL, in 12-well, 6 droplets of 20 μL, and in 6-well, 10 droplets of 20 μL.

**CRITICAL:** Thaw the BME at 4°C for 12–16 h and keep it at 4°C throughout the procedure to maintain it in liquid form.

**CRITICAL:** Prewarm the multiwell plates in the 37°C incubator for 12–16 h to ensure that the droplets solidify correctly.

***Note:*** Adjust the BME volume according to the amount of tissue digested. As a reference, 1 mm^3^ of viable tissue typically yields enough cells to plate 3–6 20 μL droplets.

10. Carefully turn the plate upside down and allow it to solidify in a 37°C incubator with 5% CO_2_ for approximately 20 min.
11. Add pre-warmed (37°C) complete culturing medium, based on the used multiwell plate (500 μL to 24-well, 1 mL to 12-well, and 2.5 mL to 6-well plate) (Figure 1C).
12. Place the plate in a 37°C incubator with 5% CO_2_. Refresh the medium every 3–4 days.

### Quality assessment after tubuloid establishment

As described by Schutgens et al.,^3^ healthy kidney tubuloids form spherical cystic structures of single-layered, polarized epithelium, which remodel over time into folded tubular structures.

Immunofluorescent markers can be used to validate the tubular epithelial identity (PAX8) and polarity (ITGA6, ZO-1 and F-actin), as well as specific segments such as proximal tubules (VIL1) and collecting ducts (AQP3).^3^

### Passaging of human kidney tubuloids

**Timing: 30–45 min**

This section describes the passaging of established tubuloid lines. We use tissue-derived tubuloids as an example, but the basic principles can be generally applied to any type of tumor and/or normal tissue-derived organoid model.

**Figure 2:**
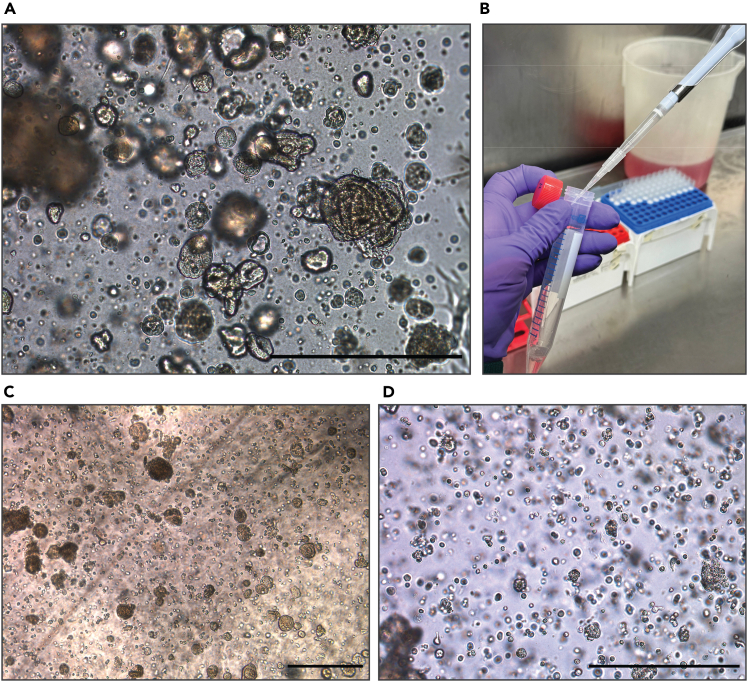
Passaging of a tubuloid culture (A) Representative brightfield image of a typical tubuloid culture at passaging. (B) Mechanical dissociation using a non-filtered p10 tip on top of a filtered p1000 pipet tip. (C) Example of partial disruption of tubuloids. (D) Single cell suspension after complete disruption of tubuloids. Scale bars = 500 μm.

***Note:*** Expansion rates can vary according to tissue/tumor type, as previously described.^2^^,^^3^^,^^7^ Typically, tubuloids can be passaged every 7 days at ratios between 1:2-1:3 splits. We recommend seeding cells within a range of 1000–2500 cells/μL of BME. An example of tubuloids ready for passaging is provided in Figure 2A.

13. Disrupt the plated droplets containing the tubuloids by pipetting up and down with a p1000 filter tip using the culturing medium present in the well. Use the tip to scrape the bottom of the well, ensuring to collect all cells that are attached to the bottom and transfer all contents to a 15 mL tube.
14. Add 10 mL of ice-cold AdvDF+++ and centrifuge at 250 x *g* for 5 min at 4°C. Remove the supernatant.
15. Add TrypLE supplemented with Rho-kinase inhibitor (10 μM) to the pellet. Use 500 μL of TrypLE per 100 μL of tubuloid containing BME. Incubate at 37°C for 5 min.
16. To mechanically disrupt the tubuloids, place a non-filtered p10 tip on top of a filtered p1000 pipette tip and pipet up and down 20 times (Figure 2B).

**CRITICAL:** Check through the microscope if many intact organoids are still present. If the majority of the tubuloids are still intact, repeat the incubation with TrypLE for 5 min at 37°C and mechanical disruption (Figures 2C and 2D).

17. Plate the cell pellet in BME.
  a. Carefully resuspend the pellet by pipetting up and down without creating air bubbles.
  b. Add 70%–75% volume of BME to the pellet.
  c. Resuspend carefully with a p200 pipette and plate 20 μL droplets in a pre-warmed multiwell cell culture plate, based on total plating volume. In 24-well, 3 droplets of 20 μL, in 12-well, 6 droplets of 20 μL, and in 6-well, 10 droplets of 20 μL.
18. Carefully turn the plate upside down and allow it to solidify in a 37°C incubator with 5% CO_2_ for approximately 20 min.
19. Add pre-warmed (37°C) complete culturing medium based on the used multiwell plate (500 μL to 24-well, 1 mL to 12-well, and 2.5 mL to 6-well plate). Check the organoids under the microscope.
20. Incubate in a 37°C incubator with 5% CO_2_. Refresh the medium every 3–4 days.

### Lentiviral transduction of established human kidney tubuloid cultures

**Timing: 1–3 weeks**

This section describes the production of lentiviral particles using HEK293T cells and the lentiviral transduction of human kidney tubuloids via spinoculation.

21. Plating HEK293T for transfection (day 1).
  a. Aspirate the culture medium from a 15 cm dish of HEK293T cells.
  b. Gently add 10 mL of DPBS to the side of the dish and swirl to wash the cells attached.
  c. Remove the DPBS and add 2 mL of pre-warmed (37°C) TrypLE.
  d. Incubate at 37°C for 2–3 min.
  e. Observe the cells under a brightfield microscope. When they are detached, add 10 mL of pre-warmed (37°C) culture medium to inactivate the TrypLE. Resuspend the cell suspension thoroughly.
  f. Plate the cell suspension in three new 15 cm dishes. Add 4 mL of cell suspension per dish (split the cells at a 1:3 ratio).
  g. Add 16 mL of culture medium per dish and swirl to distribute the cells.
  h. Incubate the plates in a 37°C incubator with 5% CO_2_ until the day after (or until they have reached a confluency of approximately 75% at the day of transfection).

***Note:*** Plate one 15 cm dish per virus production. If your construct does not include a fluorescent marker, we recommend seeding an additional control plate and transfecting it with a construct expressing a fluorescent protein. This will provide a readout for transfection efficiency.

22. Transfecting HEK293T for lentivirus production (day 2).
  ***Note:*** Cells should be 70%–80% confluent to maximize the transfection efficiency.
  a. Prepare a OptiMEM-DNA mix as described in Table 1.
    ***Note:*** The quantities of DNA depicted are per 15 cm culture dish.

**Table 1:** Plasmids and chemicals for the transfection mix

Reagent	Amount
OptiMEM	3 mL
pHDM-VSV-G	7.2 μg
pRC/CMV-Rev1b	3.6 μg
pHDM-Tat1b	3.6 μg
pHDM-Hgpm2	3.6 μg
Plasmid of interest	45 μg
  b. Vortex for 2 s at low speed (set to level 3 on a Vortex-Genie 2 mixer).
  c. Prepare an OptiMEM-PEI mix containing 2 mL of OptiMEM and 315 μL of PEI.
    ***Note:*** The amount of PEI (in μL) corresponds to 5 times the amount of DNA (in μg).
  d. Vortex for 2 s at low speed (set to level 3 on a Vortex-Genie 2 mixer).
  e. Incubate the OptiMEM-PEI mix for 5 min at 18°C–25°C.
  f. Dropwise, add the OptiMEM-PEI mix to the OptiMEM-DNA mix, creating a PEI/DNA mix.
  g. Vortex for 2 s at low speed (set to level 3 on a Vortex-Genie 2 mixer).
  h. Incubate the PEI/DNA mix for 30 min at 18°C–25°C.
  i. Add the PEI/DNA mixture to the cells dropwise.
  j. Gently tilt the plate to mix PEI/DNA with the medium in the plate.
  k. Incubate the plates in a 37°C incubator with 5% CO_2_ for 18–24 h.
23. Checking transfection efficiency and refreshing medium (day 3).
  a. One day after transfection, check the expression of the fluorescent marker in your plates under the microscope. Alternatively, assess the transfection efficiency of the control plate. If successful, proceed with the protocol.
  b. Remove the culture medium.
  c. Carefully add 10 mL of pre-warmed (37°C) culture medium by pipetting at the side of the plate (to avoid detachment of cells).
  d. Incubate the plates in a 37°C incubator with 5% CO_2_ for 2 days.
24. Harvesting and concentrating the virus (day 5).
  a. Collect the virus-containing medium from the 15 cm dishes. Be careful not to touch the adherent cells.
  b. Filter the medium with a 0.45 μm syringe filter.
  c. Pour the filtered medium into ultracentrifuge tubes.
    **CRITICAL:** Ensure that the ultracentrifuge tubes are balanced with a maximum difference of 0.01 g using culture medium.
  d. Spin down the tubes for 1 h 30 min at 4°C at 20000 *g* using a fixed angle rotor (e.g., Rotor type 50.2 Ti).
    ***Note:*** If the ultracentrifuge is not available, use an Eppendorf centrifuge 5810R with fixed-angle rotor FA-45-6-30 and 50 mL centrifuge tubes suitable for high-speed rotors.
  e. After centrifugation, carefully remove the supernatant.
    **CRITICAL:** After centrifugation the pellet might be hardly visible. Mark the tube in advance with a circle where you expect the pellet to form and aspirate the supernatant from the opposite side.
  f. Resuspend the viral pellet in 500 μL of organoid culture medium.
  g. Aliquot the virus and snap freeze in liquid nitrogen. Store the aliquots at −80°C.
    **CRITICAL:** Prepare aliquots to avoid repeated freeze-thaw cycles, which can reduce the viral titer. Adjust the volume of each aliquot based on your experimental plan.
25. Lentiviral transduction of tubuloids via spinoculation (day 6).
  a. Collect and dissociate the tubuloids into a single cell suspension as explained in steps 13–16.
    ***Note:*** Tubuloids should be in their proliferative phase (4-6 days after the last split, depending on the model).
  b. Count the cells and assess their viability with Trypan Blue staining.
  c. Harvest the desired amount of cells for transduction and transfer to a 15 mL tube. Include cells to use as untransduced control.
  d. Fill up the tube with ice-cold AdvDF+++ and centrifuge at 250 × *g* for 5 min at 4°C.
  e. Remove the supernatant and resuspend the pellet in medium supplemented with polybrene 10 μg/mL. Calculate 25 μL of medium per reaction.
  f. For each reaction, transfer 25 μL of cell suspension in a well of a pre-warmed (37°C) plate. The size of the plate depends on the number of cells to transduce, as explained in Table 2.

**Table 2:** Cell numbers and volumes of transduction

Cell number per reaction	Reaction volume	Cell culture plate
1∗10ˆ6	250 μL	24-well plate (one well)
0.5∗10ˆ6	125 μL	48-well plate (one well)
0.1–0.25∗10ˆ6	70 μL	96-well plate (one well)
  g. Add the desired amount of lentivirus in each well and adjust the reaction volume using culturing medium supplemented with Polybrene 10 μg/mL. Leave one well without virus as control for the transduction.
    ***Note:*** For high transduction efficiency (above 75%), add 20–25 μL of ultracentrifuged virus to 1∗10ˆ6 cells. This volume was empirically optimized across multiple experiments by quantifying the fraction of cells expressing a fluorescent reporter. All lentiviral preparations were produced and concentrated using the same protocol to ensure comparable relative viral input across conditions. The optimal volume may vary between virus production batches and organoid models.
  h. Mix virus and cell suspension thoroughly without making air bubbles.
  i. Wrap the plate with parafilm.
  j. Centrifuge at 32°C for 1 h at 600 x *g* (set brake to 3).
  k. Remove parafilm and keep the plate in a 37°C incubator with 5% CO_2_ for at least 4 h.
    ***Note:*** Longer incubation (up to 6 h) is recommended for enhanced transduction efficiency.
  l. After incubation, transfer the cell-virus suspension in a 15 mL tube and flush the well with AdvDF+++.
  m. Fill up the tube with ice-cold AdvDF+++ and centrifuge at 250 × *g* for 5 min at 4°C.
  n. Remove the medium, resuspend the cells in BME and seed in a pre-warmed multi-well plate as explained in steps 17–20.
26. Selection of transduced tubuloids and generation of stable transduced lines.
  a. 2-3 days after transduction, refresh the culture medium and add the selection antibiotic (example in Table 3).

**Table 3:** Recommended antibiotic concentration for selection and maintenance of transduced tubuloids

Antibiotic	Concentration
Puromycin	1 μg/mL
Blasticidin	10 μg/mL
Hygromycin	200 μg/mL
Neomycin	300 μg/mL
  b. Keep the tubuloids under antibiotic selection. Always check that the untransduced tubuloids do not survive the selection (it should take maximum 2 weeks, depending on the antibiotic).

**Figure 3:**
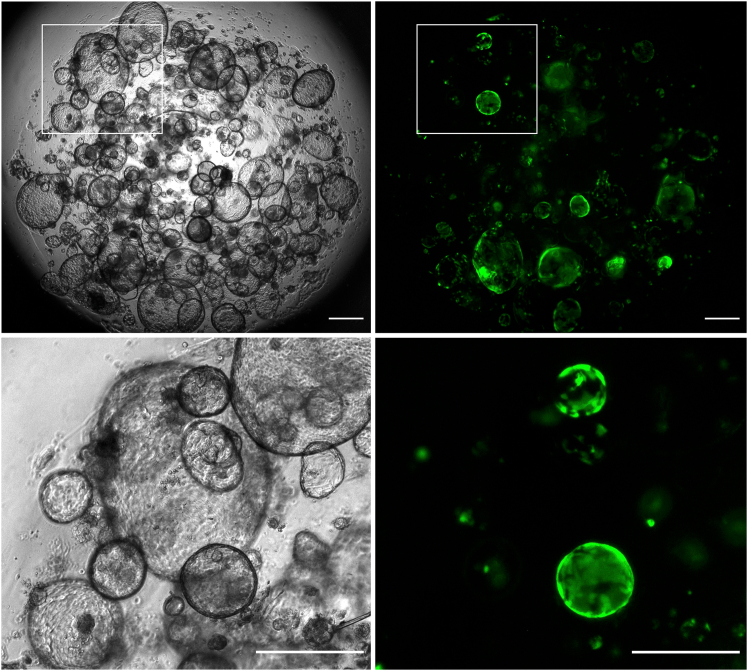
Tubuloids can be efficiently transduced using lentiviruses Brightfield (left) and GFP channel (right). Enlarged views of selected ROIs are shown at the bottom. Scale bars = 500 μm.

An example of how the transduced tubuloids should look like is provided in Figure 3.

### Formalin fixation and paraffin embedding of human kidney tubuloids for histological characterization

**Timing: 2 days**

The section below describes how to fix tubuloids in formalin and embed them in paraffin for histological analysis. The protocol is easily adaptable to organoid models derived from tumor or normal tissue, and both mouse and human tissue samples.

27. Harvest the tubuloids and fix in PFA.
  a. Collect the plated droplets containing the tubuloids as explained in step 13.
  b. Add 10 mL of ice-cold AdvDF+++ and centrifuge at 500 x *g* for 5 min at 4°C. Remove the supernatant.
  c. Add 2.5 mL of 4% PFA to 100 μL of collected BME droplets and incubate vertically for 12–16 h at 4°C (Figure 4A).
    **CRITICAL:** Incubate the organoids vertically to prevent them from adhering to the sides of the tube, which may result in sample loss in later steps.

**Figure 4:**
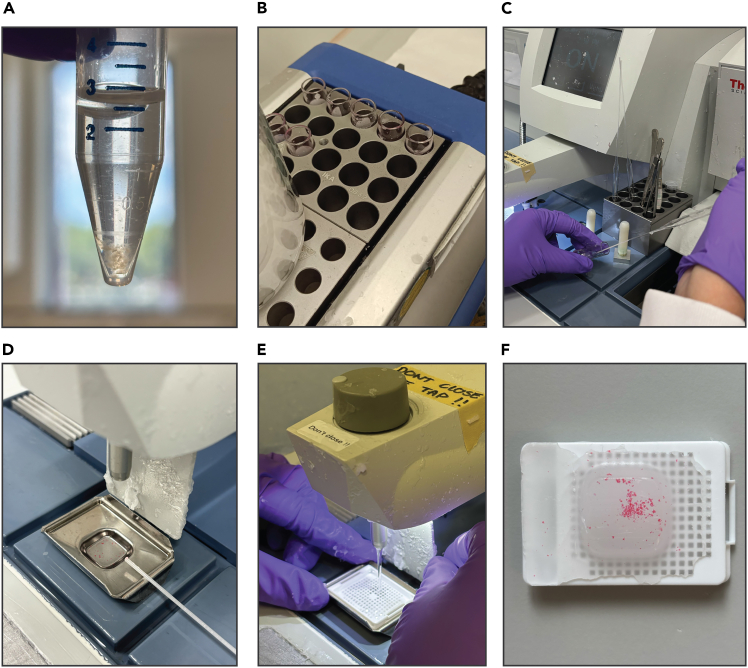
Workflow for fixation and embedding of tubuloids in paraffin (A) Fix the tubuloids in PFA. (B) Wash the tubuloids in liquid paraffin. (C) Use a glass pipette to transfer the tubuloids to a metal mold. D) Add paraffin to the metal mold. E) Place the cassette on top of the metal mold and fill with paraffin. F) Embedded tubuloids after paraffin solidification.
  d. Centrifuge at 200 x *g* for 5 min at 4°C and remove the supernatant.
    **CRITICAL:** Centrifuge at this speed to avoid clumping.
  e. Add 5 mL of PBS 1X to the pellet and incubate for 15 min at 18°C–25°C.
  f. Centrifuge at 200 x *g* for 5 min at 4°C and remove the supernatant.
  g. Repeat steps e-f two additional times.
  h. Add 5 mL of 70% ethanol to the pellet, transfer to a flat bottom glass vials and incubate for 12–16 h at 4°C.
    ***Note:*** Samples can be stored in 70% ethanol at 4°C for multiple weeks.
28. Dehydrate the fixed tubuloids through graded ethanol series.
  a. Carefully remove the 70% ethanol with 1 mL pipette tip and add 2 mL of pure eosin (ethanol-based) to the fixed tubuloids. Incubate for 10 min at 18°C–25°C.
    ***Note:*** Eosin staining facilitates the visualization of organoids in the paraffin block. The staining is subsequently removed during the deparaffinization step.
    **CRITICAL:** Before removing any solution, make sure that tubuloids are precipitated to the bottom.
  b. Carefully remove the pure eosin with 1 mL pipette tip and add 2 mL of 100% ethanol. Incubate for 30 min at 18°C–25°C.
  c. Repeat step b two additional times.
  d. Carefully remove the 100% ethanol with 1 mL pipette tip and add 2 mL of butanol. Incubate for 30 min at 18°C–25°C.
  e. Repeat step d two additional times.
29. Embed the fixed tubuloids in paraffin.
  a. Carefully remove the butanol with 1 mL pipette tip.
  b. Using a heated paraffin embedding station, fill the glass vial with liquid paraffin. Incubate for 30 min at 60°C in the heating block provided (Figure 4B). Remove the paraffin.
    **CRITICAL:** Add paraffin slowly to the tubuloids.
  c. Repeat step g two additional times. The last time, discard 75% of the paraffin.
  d. Pre-warm a glass pipette and a metal mold (60°C–65°C). Use the glass pipette to transfer the tubuloids from the glass vial to the metal mold (Figure 4C).
    **CRITICAL:** Be fast to avoid that the paraffin solidifies in the pipette. Check “Troubleshooting” section (Problem 4) for tips/alternatives.
  e. Position the tubuloids at the center of the mold.
  f. Cool down the metal mold on the cold plate of the embedding station for 10 s.
  g. Fill the metal mold with liquid paraffin (Figure 4D).
  h. Place the plastic cassette on top of the metal mold.
  i. Cover the cassette with liquid paraffin (Figure 4E).
  j. Place the cassette on the cold plate for at least 2 h to ensure complete paraffin solidification.
  k. Remove the metal mold and store the paraffin blocks at 18°C–25°C (Figure 4F).

### Histological and immunohistochemical staining of tubuloids

**Timing: 2 days**

**Figure 5:**
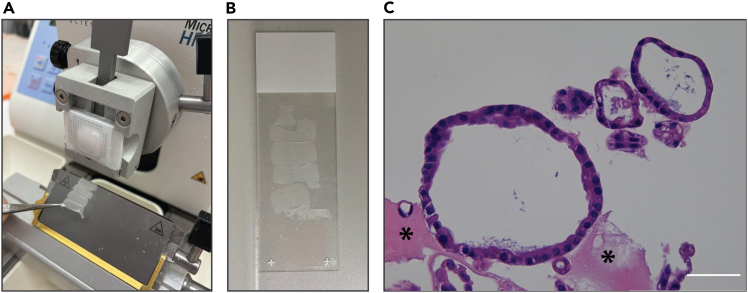
H&E staining for histological characterization of tubuloids (A) Microtome sectioning of an FFPE block. (B) Glass slide with paraffin sections. (C) H&E staining of tubuloids. (∗) Residual basement membrane extract (BME). Scale bar = 100 μm.

The section below describes the preparation and processing of FFPE slides for either hematoxylin-eosin (H&E) staining or immunohistochemistry (IHC). Steps 30 and 31 – sectioning, deparaffinization and rehydration – are common to both protocols. For H&E staining follow step 32, for IHC go to step 33 (Figure 5).

***Note:*** All incubation steps are performed in plastic buckets. Change buckets at each step.

30. Section the paraffin block using a microtome.
  a. Place the paraffin block on a cold plate for around 10 min before cutting.
  b. Cut 4 μm slices using a microtome and transfer them to a 42°C pre-warmed water bath using pre-cooled tweezers.
  c. Use a glass slide to fish out the cut slices from the water bath.
  d. Dry the cut sections for 12–16 h at 37°C to remove the water.
  e. Bake the slides for 1 h at 60°C.
31. Deparaffinize and rehydrate the section through graded ethanol series.
  a. Incubate the section for 5 min in Xylene 2 times.
  b. Incubate the section for 1 min in 100% ethanol 2 times.
  c. Incubate the section for 1 min in 96% ethanol 2 times.
  d. Incubate the section for 1 min in 80% ethanol 2 times.
  e. Incubate the section for 1 min in 70% ethanol 2 times.
  f. Incubate the section for 1 min in 50% ethanol 2 times.
32. Perform H&E staining.
  a. Incubate the section in haematoxylin solution for 2 min and rinse in tap water for 10 min.
  b. Incubate the section with tap water for 5 min.
  c. Incubate the section in eosin solution for 30 s.
  d. Perform dehydration to xylene:
    i. Incubate the section for 1 min in 80% ethanol.
    ii. Incubate the section for 1 min in 95% ethanol 2 times.
    iii. Incubate the section for 1 min in 100% ethanol 2 times.
    iv. Incubate the section for 5 min in Xylene 2 times.
  e. Enclose slides with Pertex.
33. Perform IHC staining.
  a. Leave the slides for 5 min in demi water.
  b. Incubate the slides in PO-blocking buffer for 15 min at 18°C–25°C to block endogenous peroxidase.
    **CRITICAL:** Add fresh hydrogen peroxide to the blocking buffer before use.
  c. Wash the slides in demi water for 5 min.
  d. Perform heat induced antigen retrieval by placing the slides for 20 min in boiling antigen retrieval buffer based on the target antibody.
    **CRITICAL:** Make sure to use the buffer that is compatible with the primary antibody, as recommended by the manufacturer. Check section “Materials and equipment” for the recipe of Citrate buffer pH 6.0 and Tris/EDTA buffer pH 9.0.
  e. Allow slides to cool down to 18°C–25°C in antigen retrieval buffer.
  f. Wash the slides 3 times in PBS 1X, incubating 5 min per wash and replacing the solution after each wash.
  g. Perform the blocking by adding 200 μL of 1% BSA/PBS 1X per slide. Incubate for 1 h at 18°C–25°C and cover with plastic coverslip.
    **CRITICAL:** To prevent evaporation, place pre-wetted tissues in the container to maintain a humid environment.
  h. Incubate the slides with 125 μL of primary antibody diluted in 0.5% BSA/PBS 1X at 4°C for the duration recommended by the manufacturer (typically for 12-16 h). Cover the entire slide evenly using plastic coverslips.
    NB: BSA can be substituted with serum compatible with the primary antibody (e.g., normal goat serum if using a goat-derived primary antibody).
  i. Wash the slides 3 times in PBS 1X, incubating 5 min per wash and replacing the solution after each wash.
  j. Incubate the slides with 125 μL of secondary antibody diluted in 0.5% BSA/PBS 1X for 1 h at 18°C–25°C and close with plastic coverslips.
  k. Wash slides 3 times in PBS 1X, incubating 5 min per wash and replacing the solution after each wash.
  l. Add 150 μL of DAB solution 1X per slide and incubate at room temperate for 15 min.
    **CRITICAL:** Work in a fume hood because DAB is toxic.
    ***Note:*** The exact time of DAB incubation may vary between antibodies. Check the slide under the microscope and stop the reaction when the optimal staining is achieved. We recommend using a negative control to check for the specificity of the staining.
  m. Stop DAB reaction by washing slides with demi water for 5 min at 18°C–25°C.
  n. Perform haematoxylin counterstain by dipping the slides for 4–5 s, then wash with running tap water for 1 min.
  o. Incubate the slides in acidic ethanol for 4 dips of 2 s.
  p. Wash slides for 5 min in running tap water.
  q. Perform dehydration to xylene:
    i. Incubate the section for 1 min in 25% ethanol.
    ii. Incubate the section for 1 min in 50% ethanol.
    iii. Incubate the section for 1 min in 70% ethanol.
    iv. Incubate the section for 1 min in 95% ethanol 2 times.
    v. Incubate the section for 1 min in 100% ethanol 2 times.
    vi. Incubate the section for 5 min in Xylene 2 times.
  r. Enclose slides with Pertex.

### Preparing (transduced) tubuloids for downstream omics analyses

**Timing: 2–3 days**

The section below describes the preparation of transduced tubuloids for CUT&RUN and single-cell RNA sequencing (scRNA-seq). In Ganpat et al,^1^ the 10X Genomics platform was used. However, the sample preparation also applies to other sequencing platforms.

### Preparing (transduced) tubuloids for CUT&RUN

**Timing: 2 days**

34. Collect the tubuloids and dissociate them into single cells as described in steps 13-16.
35. Wash the single cell suspension 2 times with DPBS 1X.

***Note:*** Keep the cell concentration at or below 2∗10ˆ6 cells/mL.

**CRITICAL:** All steps from now on should be performed on ice.

36. Wash the cell pellet 2 times with wash buffer 1 and resuspend in a final volume of 200 μL.

***Note:*** All washes from this step onwards should be performed at 500 × *g* for 4 min at 4°C.

**CRITICAL:** Use Protein LoBind 0.5 mL tubes.

37. Prepare wash buffer 1 containing the primary antibody at 2X the final desired dilution (200 μL per sample). Add 200 μL of primary antibody dilution to the cells for a total sample volume of 400 μL. Mix well by pipetting.

***Note:*** The antibody concentration to be used must be optimized experimentally for every individual antibody. For example, in Ganpat & Morales-Rodriguez et al.,^1^ the authors used 0.2 μg/mL of anti-TFE3 antibody (Abcam, ab93808) or for anti-H3K4Me3 antibody (Invitrogen, MA5-11199) by diluting them 1:100 or 1:500, respectively.

**CRITICAL:** For each experiment, add a negative (e.g., without any primary antibody or an isotype control), and a positive control (e.g., anti- H3K4me3, which is expected to give a robust signal at promoters).

38. Mix for 12–16 h at 4°C on roller bank.
39. Wash the samples once with 500 μL of cold wash buffer 2.
40. Resuspend the cells in 500 μL of wash buffer 2 containing ProteinA-MN (0.3 ng/mL) and Hoechst 34580 (5 μg/mL).
41. Mix for 1 h at 4°C on a roller bank.
42. Wash the samples 2 times with 500 μL of cold wash buffer 2 and resuspend the cells in 200 μL of wash buffer 2.
43. Filter the sample in a FACS tube with a blue mesh cap (40 μm mesh filter).

**CRITICAL:** Pre-wet the mesh filter with 50 μL of wash buffer 2 to decrease the stickiness to the filter.

44. Prepare a FACS collection tube by adding 5 μL of wash buffer 3 to a DNA LoBind 1.5 mL tube.
45. Sort the nuclei based on Hoechst positivity.
46. Activate ProteinA-MN by adding 5 μL of wash buffer 3 containing 4 mM Calcium chloride in a 1:1 ratio of sorted nuclei.
47. Perform the digestion by incubating the sample for 30 min at 4°C.
48. Stop the digestion.
  a. Add 10 μL of stop solution.
  b. Incubate for 6 h at 65°C, followed by 20 min at 80°C and hold at 4°C in a thermocycler.
  c. Store the samples at −20°C.

### Preparing (transduced) tubuloids for single-cell RNA sequencing

**Timing: 2 h**

49. Collect the tubuloids and dissociate them into single cells as described in steps 13–16.
50. Resuspend the cell pellet in FACS buffer. Use 500 μL of buffer for 1∗10ˆ6 cells.
51. Filter the sample in an Eppendorf tube using a 70 μm mesh filter.

**CRITICAL:** Pre-wet the mesh filter with 100 μL of FACS buffer. Push the cell suspension against the wall of the filter without touching the membrane.

52. Prepare a FACS collection tube by adding 50 μL of FACS buffer in a 1.5 mL Eppendorf tube.

**CRITICAL:** Pre-rinse the tube with 1 mL of FACS buffer to prevent the sorted cells from sticking to the side of the tube.

53. Add Draq5 (500 nM) and Dapi (1 μM) to the cell suspension and incubate for 1 min on ice.
54. Sort live cells based on Draq5 positivity and DAPI negativity.

***Note:*** If the FACS instrument uses disposable chip-based technology, use the 100 μm chip for sorting tubuloids.

55. After sorting, wash the wells of the collection tube with additional FACS buffer and centrifuge at 250 x *g* for 5 min at 4°C.
56. Remove the supernatant up to 20 μL.
57. Count the cells and assess their viability with Trypan Blue staining.
58. Collect the desired number of cells to load on a chip, as specified in the manufacturer’s protocol.
59. Continue with the downstream protocol for chip loading.

## Expected outcomes

This protocol outlines the generation and establishment of patient-derived kidney organoids, referred to as tubuloids, followed by lentiviral transduction for stable genomic integration and expression of target genes. The transduced tubuloids can subsequently be characterized through a variety of molecular and cellular assays.

This workflow enables the systematic investigation of phenotypic and transcriptional consequences of genetic modifications within a patient-derived organoid context. As demonstrated in previous studies,^1^^,^^2^^,^^3^^,^^7^^,^^8^ this platform offers a robust and physiologically relevant model for exploring tumor biology and identifying therapeutic vulnerabilities.

## Limitations

The establishment and expansion of tubuloid cultures typically require several weeks before they are suitable for downstream applications. This timeframe should be factored into experimental planning to ensure the timely execution of subsequent steps. Notably, we have observed a gradual decline in the proliferative capacity of tubuloids after approximately 15 passages post-establishment. To maintain optimal growth and experimental consistency, we recommend performing lentiviral transductions at early passages, ideally between passages 5 and 6.

This protocol does not include a viral titration step. For experiments requiring defined transduction efficiencies (e.g., requiring a certain MOI), we advise performing a titration assay in advance to determine the optimal viral concentration for the specific experimental conditions.

## Troubleshooting

### Problem 1

Incomplete dissociation of tubuloids following enzymatic disruption (Step 16).

### Potential solution

Extend the incubation time with TrypLE by an additional 5 min, followed by mechanical trituration to aid in dissociation. Alternatively, use an autoclaved glass Pasteur pipette after TrypLE treatment to enhance mechanical disruption. Complete dissociation of all tubuloids may not be feasible without compromising cell viability. If most of the organoids are disrupted into single cells or small clusters, the dissociation can be considered successful.

### Problem 2

Absence of visible virus pellet after ultracentrifugation (Step 24e).

### Potential solution

Increase the starting volume of virus-containing medium per ultracentrifugation tube to improve viral yield. Pooling supernatants from multiple 15 cm dishes (e.g., two instead of one) before ultracentrifugation can further enhance viral concentration. Additionally, use a construct expressing a fluorescent reporter to monitor transfection and transduction efficiency.

### Problem 3

Inefficient lentiviral transduction of tubuloids (Step 25).

### Potential solution

Ensure that tubuloids are in an active proliferative state, typically 4–6 days post-passaging, depending on the model. Increase the quantity of lentiviral particles used during transduction to boost efficiency. Following spinoculation, allow cells to recover for up to 8 h before changing the medium. If cells appear stressed 48 h post-transduction, delay antibiotic selection until 72 h post-transduction.

### Problem 4

Paraffin hardens in the glass pipette while transferring the organoids to the mold (Step 29i).

### Potential solution

As an alternative to glass pipette for transfering the organoids, solidify the paraffin with tubuloids directly in the glass vial by placing it on the cold plate of the embedding station. Once solidified, use the tweezers to transfer the paraffin onto the metal mold. Melt the paraffin on the heating block before proceeding with embedding.

### Problem 5

Low viability or cell number after FACS sorting for single-cell RNA sequencing (Step 57).

### Potential solution

• If starting from a cryopreserved stock, thaw cells rapidly and add culture medium dropwise to a 15 mL tube containing the thawed cells; avoid adding thawed cells directly into a pre-filled tube.
• Keep cells on ice during all waiting steps to minimize stress.
• During filtration, take care not to contact or disrupt the membrane of the cell strainer to prevent cell loss.

## Resource availability

### Lead contact

Further information and requests for resources and reagents should be directed to and will be fulfilled by the lead contact, Jarno Drost (j.drost@prinsesmaximacentrum.nl).

### Technical contact

Technical questions on executing this protocol should be directed to and will be answered by the technical contact, Jarno Drost (j.drost@prinsesmaximacentrum.nl).

### Materials availability

This study did not generate new unique reagents.

### Data and code availability

This paper did not generate new datasets.

## Acknowledgments

We are grateful for the support of the European Research Council (ERC) starting grant 850571 (J.D.); Oncode Institute, which is partly financed by the Dutch Cancer Society (J.D.); Oncode Accelerator, a Dutch National Growth Fund project under grant number NGFOP2201 (J.D.); and Foundation Children Cancer-free (KiKa, core funding).

## Author contributions

G.P. performed the experimental work. M.M.P.G. and G.P. developed and optimized the protocol. J.D. supervised the research. G.P. and J.D. wrote the manuscript.

## Declaration of interests

The authors declare no competing interests.

## Declaration of generative AI and AI-assisted technologies in the writing process

During the preparation of this work, the authors used ChatGPT to proofread the manuscript for readability and language. After using this tool, the authors reviewed and edited the content as needed and take full responsibility for the content of the published article.
